# Self-Esteem, Attitudes toward Love, and Sexual Assertiveness among Pregnant Adolescents

**DOI:** 10.3390/ijerph18031270

**Published:** 2021-01-31

**Authors:** Nieves Moyano, Reina Granados, Christian Andrés Durán, Carlos Galarza

**Affiliations:** 1 Department of Evolutionary and Education Psychology, Faculty of Humanities and Sciences Education, University of Jaén, 23071 Jaén, Spain; mnmoyano@ujaen.es; 2 Department of Nursey, Faculty of Health Sciences, University of Granada, 18071 Granada, Spain; 3 Independent Researchers, Guayaquil, Ecuador; cduranr@uees.edu.ec (C.A.D.); galarza_carloss@hotmail.com (C.G.)

**Keywords:** self-esteem, attitudes toward love, sexual assertiveness, pregnancy, adolescent

## Abstract

Adolescence is a stage of growth and development of great relevance. Unplanned teenage pregnancies can be considered a global public health problem due to the high impact on the present and future of these young people, as well as their possible offspring. The aim of this study was to analyze the relationship between self-esteem, attitudes toward love, and sexual assertiveness among pregnant and non-pregnant teenagers. We also considered whether their pregnancy was planned or not. The study was conducted with 225 women from Ecuador (34.2% pregnant; Mean age = 16; SD = 1.15). We administered self-reported measures such as the Rosenberg Self-Esteem Scale, the Love Attitudes Scale, and the Sexual Assertiveness Scale between 2018 and 2019. Self-esteem was higher in adolescents with a planned pregnancy than in those women whose pregnancy was not planned. Pregnant women reported greater acceptance and endorsement of beliefs related to the myth of “soulmate” in comparison to non-pregnant women. Sexual assertiveness related to the negotiation of the use of contraceptive methods was greater in non-pregnant adolescents than in pregnant girls. We discuss the implications of our findings in terms of sexual education and prevention in the sex education field. This study shows differences in self-esteem, attitudes toward love, and sexual assertiveness between pregnant and non-pregnant adolescents.

## 1. Introduction

Adolescence is a stage of growth and development of great relevance [1]. This period is not only characterized by biological changes but also by psychological, emotional, behavioral, and social transformations [2]. These changes and the pressures exerted by peer groups are likely to give rise to an increase in vulnerability in terms of risky behaviors, such as the consumption of tobacco, alcohol, and other drugs and early initiation in sexual relations, among others [1,3].

At this stage, challenges of a sexual nature take on a special value. According to the WHO [1], at the onset of adolescence, adolescents are not fully capable of understanding the relationship between a behavior and its consequences, or of perceiving their ability to judge when making decisions related to their health. Thus, adolescents frequently perform risky sexual behaviors, that is, behaviors that increase the probability of suffering negative consequences derived from sexual activity. These consequences can include infections by the human immunodeficiency virus (HIV), other sexually transmitted infections (STIs), and unplanned pregnancies [4].

In relation to unplanned pregnancies, the WHO establishes adolescent pregnancy as between 10 and 19 years of age [1]. Globally, 2.1 million unplanned births take place each year, as well as 3.2 million abortions, and 5,600 maternal deaths in women between the ages of 15 and 19 [5]. Likewise, from 2010 to 2015, there was an average of 46.7 births for every 1000 women in the world (age range = 15–19 years), reaching a rate of 68.1 births in Latin America and the Caribbean for every 1000 adolescents of the same age [6]. In this context, the forecast established by the United Nations between 2015 and 2020 will be 63 births in adolescent women, again exceeding the world rate (i.e., 42.5 births) [6].

In Latin America, the statistics of Ecuador stand out, as it ranks as one of the five Latin American countries (i.e., Nicaragua, Honduras, Panama, and Guatemala) with the highest rate of early pregnancy [7]. According to data published by the National Institute of Statistics and Censuses of Ecuador [8] in 2010, there were 122,301 mothers between the ages of 12 and 19, that is, 107 for every 1000 pubescent women. Specifically, in the Ecuadorian city of Guayaquil, in a study with 300 adolescents between the ages of 13 and 16 of both sexes, the most alluded reasons for the maintenance and increase of early pregnancies were “because they don’t care” and “laziness to use a condom” [9]. Furthermore, it was observed that, although 87% of the respondents indicated having information about contraceptive methods, 45% revealed having had sexual intercourse, and only 35% had ever used this type of method. In the same city, Orozco-Dávila [10] observed that 6% of the sexually active adolescent women surveyed had not heard about any contraceptive method, and those who knew of their existence did not present an adequate level of knowledge. Along these lines, 19% indicated that they had never used contraceptive methods, which led to unplanned pregnancies. Given the latter, the National Plan for Good Living in Ecuador (2013–2017) [11] set the objective of reducing teenage pregnancies in the country. According to this national plan, teenage pregnancies are caused by a lack of access to sexual and reproductive health education [11].

Due to the problems related to unwanted and/or planned pregnancy (e.g., risk of death for the mother and the fetus, poverty, family instability, school dropout, and precarious insertion in the labor market, among others) [11,12,13], it is essential to examine the factors associated with this phenomenon. Low self-esteem is one of the variables linked to early and unprotected sexual activity [14]. According to Rosenthal and Simeonsson [15], people with low self-esteem have changing identities and are unstable and openly vulnerable to criticism or rejection, thus substantiating their perceived inadequacy, incompetence, and lack of worth. Low self-esteem is the most prominent and evidenced factor in increased risk of teenage pregnancies since it is related to insecurity, fear of being rejected, low ambitions, or insufficient emotional control [16]. By contrast, adolescents with high self-esteem tend to present greater knowledge of sexual education, thus preventing them from maintaining risky sexual behaviors [17].

Another factor associated with teenage pregnancy is the idea of “romantic love” [18], that is, the belief that love is the only thing that gives meaning to life. Within this conceptualization of love, women tend to take a passive stance and tend to idealize some of the stereotyped beliefs and roles associated with men and women [19]. In puberty, this type of love predominates, thus provoking intense emotional reactions and the idealization of couple relationships, which can trigger risky sexual behaviors [20].

Finally, it is not possible to address sexual behavior without considering constructs that are specifically related to sexuality, such as sexual assertiveness. Sexual assertiveness is defined as the voluntary ability to initiate or reject unwanted sexual activities, as well as to negotiate the use of contraceptive/barrier methods to perform sexual behaviors in the healthiest and most satisfactory way [21]. Despite the relevance of this construct, most studies have focused on the role that sexual assertiveness plays in risky sexual behaviors [22], specifically those that may lead to STI transmission, with few studies focusing on behaviors that favor the risk of teenage pregnancy.

Based on the above, unplanned teenage pregnancies can be considered a global public health problem due to the high impact on the present and future of these young people, as well as their possible offspring [13]. Thus, the present study aims to examine individual factors involved in teenage pregnancy. To do this, the relationship between self-esteem, attitudes toward love, and sexual assertiveness is analyzed through a comparative analysis between pregnant (distinguishing between those who planned their pregnancy or not) and non-pregnant adolescents. Finally, those who are sexually active are examined, comparing the group of pregnant adolescents with the group of non-pregnant adolescents. To this end, the following hypotheses were tested:

**Hypothesis** **1.** *Pregnant adolescents will report less self-esteem, greater attitudes toward love, in particular more love-related myths, and less sexual assertiveness in negotiating contraception compared to the group of non-pregnant adolescents.*

**Hypothesis** **2.** *Pregnant adolescents who had planned their pregnancies will report higher self-esteem, lower attitudes toward love, that is, fewer love-related myths, and greater sexual assertiveness in negotiating contraception compared to the group of pregnant women who had not planned their pregnancies.*

**Hypothesis** **3.** *Non-pregnant adolescents who maintain an active sex life, compared to pregnant teenagers, will report higher self-esteem, lower attitudes toward love, in particular, fewer love-related myths, and increased sexual assertiveness in negotiating contraceptive methods. The introduction should briefly place the study in a broad context and highlight why it is important.*

## 2. Materials and Methods 

### 2.1. Participants

The total sample consisted of 225 women aged between 14 and 20 years old (M = 16; SD = 1.15) from Guayaquil, Ecuador. Data were collected between 2018 and 2019. The sample was classified according to its characteristics as follows: (a) Group 1 (pregnant) (*n* = 67), made up of participants in their gestational period (Mean age = 16.6; SD = 1.19). This group of girls was recruited from the maternity service of the Hospital Enrique C. Sotomayor (Ecuador), and (b) Group 2, made up of 158 adolescents from schools in the same city (Mean age = 15.74; SD = 1.04). According to the sociodemographic area girls were recruited from, most of them belonged to low-to-middle class. Inclusion criteria: according to the WHO criteria [1], adolescent women between 10 and 19 years of age were considered; regarding pregnancy status: pregnant women (who had or had not planned this pregnancy) and non-pregnant women. 

### 2.2. Measures

Sociodemographic questionnaire. Information was collected on age, education level, marital status, family structure, sexual activity, use of contraceptive methods, pregnancy planning, etc.

The Rosenberg Self-Esteem Scale [23,24] is made up of 10 statements referring to how individuals feel about themselves (e.g., “I am convinced that I have good qualities”), of which 5 items require inverting scores to obtain an overall score. Response options ranged from 1 (strongly disagree) to 4 (strongly agree). Higher scores indicate better self-esteem. In the present study, an acquiescence response pattern was observed, the last 5 items on the scale (inverted) being those that were answered inconsistently with respect to the first 5 (direct) items on the scale, thus not showing its reliability. Previous studies have shown the weakness of the 5 negatively worded items. For example, the factor loadings of the negative items were lower than those from the positive items [24] or by showing in some cases a two-factor structure in which most of the negatively worded items were omitted [25,26] or even the conclusion that “most cultures possess a negative item bias, tending to report lower levels of self-esteem on negatively phrased items than would be expected given their responses to positive items” (p. 637) as indicated in an study conducted in 53 countries using this scale [27]. Therefore, it was considered advisable to take into account the sum of the first five items as a measure of self-esteem. The response range in this case went from 0 to 25. Cronbach’s alpha for this study, of the first five items, was 0.83.

The reduced 18-item version in Spanish [24] of the Love Attitudes Scale [28,29] was used. This assesses attitudes toward these six styles of love: Eros (passionate love), Ludus (game-playing love), Storge (friendship love), Pragma (practical love), Mania (possessive, dependent love), and Agape (altruistic love). Response options ranged from 1 (strongly disagree) to 5 (strongly agree). Since there was no previously validated version in Ecuador, a preliminary analysis of the items was carried out with the data collected in this study, analyzing the internal consistency for each subscale, as well as the item–total correlation. Each subscale was similarly configured for the Spanish version, with Eros (items 1, 2, 3), Ludus (items 4, 5, 6), Storge (items 7, 8, 9), Pragma (items 10, 11, 12), Mania (items 13, 14, 15), and Agape (items 16, 17, 18). The Mania subscale only included items 14 and 15, since item 13 did not maintain an item–total correlation of over 0.30, and its elimination improved Cronbach’s alpha in this factor. For the global scale, it was 0.70. Higher scores indicate more positive attitudes toward each type of love. Examples of some of the items are: Eros (“I feel that my partner and I were meant for each other”), Ludus (“I have sometimes had to keep my partner from finding out about other partners”), Storge (“Our love is the best kind because it grew out of a long friendship”), Pragma (“A main consideration in choosing my partner was how he/she would reflect on my family”), Mania (“I cannot relax if I suspect that my partner is with someone else”), and Agape (“I would rather suffer myself than let my partner suffer”). In the present study, Cronbach’s alpha for each subscale was 0.72, 0.67, 0.66, 0.57, 0.60, and 0.62, respectively. For the purposes of this study, both the score from the subscales and for each particular item is considered, in order to gain greater insight into each specific love-related myth. 

The Spanish version from Sierra et al. [30], of the Sexual Assertiveness Scale, originally developed by Morokoff [21], was used. The scale consists of 18 items, distributed into three dimensions of sexual assertiveness: (1) initiation, (2) refusal, and (3) negotiation/use of condoms. However, for the purposes of this study, only the items corresponding to the negotiation/condom use dimension were applied (i.e., items 13–18), referring to the ability to negotiate the use of contraceptive methods, specifically the condom, in the context of sexual relations (specifically, the condom), e.g., “If my partner insists, I have sex without using a condom or latex barrier, even if I don’t want to”. Response options ranged from 0 (Never) to 4 (Always). Higher scores indicated greater assertiveness. In the present study, Cronbach’s alpha was equal to 0.69.

### 2.3. Procedure

Firstly, authorization was obtained from the Head of the Hospital Enrique C. Sotomayor (Ecuador) in order to administer the scales, and to use the information obtained during the interviews regarding the sociodemographic data of the women belonging to Group 1 (pregnant women). Likewise, the authorization and approval from the principal of several schools in the city of Guayaquil were obtained to be able to conduct the interviews and obtain information from Group 2 (non-pregnant). The instruments were applied by two researchers, after receiving the respective training. In all cases, the general objective of the research was explained, and the requirements and questions asked by the participants were clarified. In addition, the voluntary nature of the study was reported, clarifying that the research was for scientific purposes only.

Due to the characteristics of the sample, the application of self-reported measures was adapted for each case. In Group 1 (pregnant women), they were applied individually and privately in a separate room. Upon completion, the self-reported measures were handed over to the evaluator in a sealed envelope. To collect the Group 2 sample (non-pregnant), the measures were collectively applied, in paper–pencil format, in classrooms with enough space between them to favor and guarantee full privacy. The time required to complete the questionnaire was approximately 30 min. In all cases, the anonymity and confidentiality of their data were guaranteed. All participants provided written informed consent. Subjects gave their informed consent for inclusion before they participated in the study. The study was conducted in accordance with the Declaration of Helsinki and was previously approved by the University of Especialidades Espíritu Santo (Guayaquil, Ecuador). 

### 2.4. Statistical Analyses 

First, descriptive analyses of the sample were carried out. After this, to check if there were statistically significant differences between pregnant and non-pregnant women in the variables evaluated, the Mann–Whitney U statistic was used. With the same statistic, it was checked whether there were differences between women who had planned their pregnancy and those who had not. Similarly, it was examined whether there were statistically significant differences between the pregnant group and sexually active non-pregnant women. All statistical analyses were performed by SPSS v.22 (IBM Corp., Armonk, NY, USA).

## 3. Results

### 3.1. Pregnant vs. Non-Pregnant Adolescents

In general, in the group of pregnant adolescents, the majority had a stable partner—more specifically, 65% in the planned pregnancy group and 50% in the unplanned pregnancy group. Regarding their education, most had primary studies, that is, 59% and 50% in the planned and unplanned pregnancy group, respectively. In the group of 158 non-pregnant adolescents, 52 of them (32.9%) indicated having had sexual intercourse, while 106 had not (67%). Then, we analyzed whether there were significant differences between pregnant and non-pregnant adolescents in self-esteem, attitudes toward love, and assertiveness for negotiating the use of condoms. For this, the Mann–Whitney non-parametric test was carried out, considering the sample size. Table 1 shows the descriptive statistics (means and standard deviations) according to group and variable. Significant differences were found in attitudes toward love, specifically in the Pragma dimension, with the group of pregnant adolescents obtaining higher scores in this type of myth compared to non-pregnant adolescents. In the detailed analysis of each attitude toward love item, significant differences were observed in items 2, 8, 11, 14, 17, and 18, with higher scores in all of them for the pregnant group, as can be seen in Table 1. Likewise, significant differences were found in assertiveness for condom negotiation, with the group of pregnant adolescents being the least assertive (M = 2.69; SD = 3.46), compared to the group of non-pregnant adolescents (M = 5.07; SD = 4.60). No significant differences in self-esteem were yielded.

### 3.2. Planned vs. Non-Planned Pregnancy

Scores in the variables examined were compared between adolescents who had planned their pregnancies (*n* = 20) and those who had not (*n* = 47). To this end, the non-parametric Mann–Whitney test was carried out. As shown in Table 2, significant differences were observed in the self-esteem variable (U Mann–Whitney = 278.00; *p* = 0.023), in such a way that the participants who had not planned their pregnancies reported having lower self-esteem (M = 15.46; SD = 4.56) than those who had decided or planned to become pregnant (M = 17.55; SD = 3.57). No significant differences were found regarding attitudes toward love or assertiveness in the negotiation of condom use.

### 3.3. Pregnant vs. Non-Pregnant Adolescents (with Active Sexual Life)

Finally, as seen in Table 3, the group of pregnant adolescents was compared, regardless of whether the pregnancy had been planned or not (*n* = 67), with the group of non-pregnant adolescents, who had had sexual relations not resulting in pregnancies (*n* = 52), in terms of self-esteem, attitudes toward love, and sexual assertiveness of negotiation. Significant differences were found in sexual assertiveness in the use of contraceptive methods (U Mann–Whitney = 1189.00, *p* = 0.01), the group of non-pregnant and sexually active adolescents obtaining higher scores for the negotiation of contraceptive methods than the group of pregnant girls.

## 4. Discussion

The present study aimed to analyze the relationship between self-esteem, attitudes toward love (myths), and sexual assertiveness in pregnant and non-pregnant adolescents, also considering the planning or not of their pregnancy. Comparisons were conducted considering pregnant adolescents, and adolescents who were not pregnant but had had sex. In general, it is noteworthy that pregnant adolescents, compared to non-pregnant adolescents—and particularly when compared to non-pregnant but sexually active adolescents—present more myths about love related to their “soulmates”. In this sense, the role played by women is one of sacrifice, as they consider that the well-being of their partners is more important than their own desires in life. There is also less ability to negotiate the use of contraceptive methods in sexual relations. On the other hand, the importance of self-esteem in aspects such as pregnancy planning is highlighted, this being higher in women who plan their pregnancy in contrast to those who do not.

Regarding the first hypothesis, pregnant adolescents were expected to present lower self-esteem and sexual assertiveness, in addition to more positive attitudes toward love compared to non-pregnant adolescents. Regarding self-esteem, the hypothesis could not be confirmed due to the absence of statistical significance. Therefore, it is likely that the differences in adolescent sexual activity are not determined solely by self-esteem, but rather, they may be due to the environment and the subject’s state of mind. On the other hand, non-pregnant adolescents indicate greater sexual assertiveness related to the negotiation and use of contraceptive/barrier methods than pregnant participants. This result was expected since sexual assertiveness is considered a protection factor against sexual behaviors that are risky or that can lead to unplanned pregnancies [22]. Following the first hypothesis, pregnant women seem to have more beliefs related to certain attitudes toward love and myths, specifically of the Pragmatic type, in contrast to non-pregnant adolescents. This result is in line with previous studies where the predominance of romantic love is established as a risk factor for early pregnancy [18]. In addition, the items from the Pragmatic dimension, and those in which statistically significant differences were found (e.g., 2, 8, 14, 17, and 18), refer to the couple, their involvement with the family, their ability to be a good mother or father, and the support that will be received from them [24], among others. In this line, especially from the last trimester of pregnancy, women tend to establish concerns related to the well-being of their future child and of themselves, as well as the imminent modification of their customs and personal goals [31]. Therefore, social support and specifically the emotional support of the couple become determining factors at this stage [32].

Teenagers who had not planned their pregnancies report lower self-esteem than those who had. Self-esteem favors people acting and finding a way to achieve their goals [16]. Therefore, our findings may indicate that these adolescents (e.g., intentional pregnancy) with high self-esteem have clearer goals and an established life plan. Conversely, low self-esteem is considered to predispose to unexpected adolescent pregnancies, so interventions in this regard should not only focus on family planning but also on psychological reinforcement [16].

Regarding the third hypothesis of the present study, this was partially confirmed since only significant results in sexual assertiveness were observed. In this case, the sexually active and non-pregnant women presented higher levels of sexual assertiveness related to the prevention of pregnancies and STIs, and to the negotiation of contraceptive methods than the pregnant young women. This assertiveness is related to less participation in risky sexual behaviors, which becomes a protective variable against this type of behavior [33].

Finally, our findings are consistent with studies conducted in other countries. Pregnancy leads to several changes in women who may experience lack of confidence, fragility, fear, and therefore low self-esteem [34]. Regarding sexual assertiveness, a systematic review or 76 studies from different countries worldwide indicates that women with low self-esteem also report inconsistently using mechanisms of birth control [22]. Therefore, sexual education programs help to improve sexual assertiveness in adolescent girls [35,36]. Finally, it is important to analyze whether adolescents engage in unprotected sexual behavior due to peer pressure, drugs/alcohol, sexual experimentation, myths about contraception, the media, or others factors such as the fear of partner rejection as recently indicated [37]. 

This study has some limitations. Our findings cannot be extrapolated to the general population because the sampling method was not random. In addition, the sample focused on Ecuadorian adolescent women, in which case the group of pregnant young women went to the same health center, which was a public one, and therefore, their sociodemographic characteristics were homogeneous. For this reason, future studies should include other age ranges, as well as cross-cultural comparisons with different populations and nationalities. Aspects not considered in this study were religious–cultural beliefs related to the termination of pregnancy. These beliefs have negative implications for the person who decides not to continue with their pregnancy, which can affect their self-esteem and their assertiveness. Likewise, it is worth highlighting the opinion of parents and their great influence on the final decisions of adolescents [38]. In future studies, it is recommended to take into account these two variables.

## 5. Conclusions

In summary, self-esteem, beliefs about love, and sexual assertiveness are factors that play a relevant role in the risk of teenage pregnancy. Specifically, women who did not plan their pregnancies have lower levels of self-esteem than those who did. Likewise, pregnant adolescents have greater beliefs in romantic myths, especially those where women idealize their male partners, and the role of the latter becomes predominant. Finally, sexual assertiveness was lower in pregnant women than in women who had not had sexual intercourse, and in others who were sexually active. These findings highlight the relevance of adequate sexual and mental health education in adolescents to avoid unwanted and/or unplanned pregnancies, as well as other consequences derived from risky sexual behaviors. Therefore, health education, and more specifically sexual health education, must take into account the reinforcement of self-esteem and sexual assertiveness in this age group. In the same way, it is necessary to apply holistic interventions (i.e., informative, educational, psychological, health, etc.) that help to demystify love based on a romantic and unreal perspective, which is still culturally preserved. Furthermore, it is necessary to study other individual psychological factors that, together with those studied herein, can provide more information about the reasons that favor the increasing number of teenage pregnancies.

## Figures and Tables

**Table 1:** Descriptive statistics and comparison in self-esteem. attitudes toward love, and sexual assertiveness between pregnant and non-pregnant adolescents.

	Pregnant Adolescents (*n* = 67)	Non-Pregnant Adolescents (*n* = 158)	
Variables	M (SD)	M (SD)	U Mann–Whitney
Self-esteem	16.12 (4.27)	16.31 (2.64)	4708.5
Attitudes toward Love			
Eros	12 (3.03)	10.96 (2.71)	4507.5
Ludus	10.96 (3.86)	7.93 (3.57)	4891
Storge	16.53 (3.40)	11.77 (3.00)	5196
Pragma	11.98 (2.71)	12.46 (2.38)	4059.50 *
Mania	13.58 (4.26)	4.56 (2.16)	4511
Agape	8.42 (2.94)	7.86 (3.08)	
2. I feel that my partner and I were meant for each other.	3.83 (1.17)	3.43 (1.12)	3918.50 **
8. Our friendship merged gradually into love over time.	4.42 (0.80)	3.81 (1.24)	3699.00 **
14. Since I’ve been in love with my partner, I’ve had trouble concentrating on anything else.	3.08 (1.11)	2.43 (1.28)	3304.00 ***
17. I cannot be happy unless I place my partner’s happiness before my own.	3.42 (1.39)	2.87 (1.36)	3978.50 **
18. I am usually willing to sacrifice my own wishes to let my partner achieve his/hers.	3.03 (1.28)	2.60 (1.37)	4063.00 *
Sexual assertiveness in the use of contraceptive methods	2.69 (3.46)	5.03 (4.69)	1189.00 **

*** *p* < 0.001; ** *p* < 0.01; * *p* < 0.05. Regarding the Love Attitudes Scale, only items showing statistical significance are shown.

**Table 2:** Descriptive statistics and comparison in self-esteem, attitudes toward love, and sexual assertiveness between planned and non-planned pregnant adolescents.

	Planned Pregnancy (*n* = 20)	Non-Planned Pregnancy (*n* = 47)	
Variables	M (SD)	M (SD)	U Mann–Whitney
Self-esteem	15.46 (4.56)	17.55 (3.57)	278.00 *
Attitudes toward Love			
Eros	11.43 (2.88)	11.50 (3.05)	427.50
Ludus	7.44 (3.00)	7.93 (3.57)	411.00
Storge	11.70 (2.80)	12.60 (3.00)	364.50
Pragma	5.51 (2.37)	4.85 (1.59)	336.50
Mania	12.90 (2.15)	12.90 (1.74)	403.00
Agape	8.46 (3.08)	8.10 (2.73)	379.00
Sexual assertiveness in the use of contraceptive methods	2.69(3.46)	5.03 (4.69)	326.50

* *p* < 0.05.

**Table 3:** Descriptive statistics and comparison in self-esteem, attitudes toward love, and sexual assertiveness between pregnant and non-pregnant women (sexually active).

	Pregnant Adolescents (*n* = 67)	Non-Pregnant Adolescents (Sexually Active) (*n* = 52)	
Variables	M (SD)	M (SD)	U Mann–Whitney
Self-esteem	16.12 (4.27)	16.32 (2.11)	1671.00
Attitudes toward Love			
Eros	12 (3.03)	11.17 (2.81)	1592.50
Ludus	10.96 (3.86)	8.78 (4.12)	1417.50
Storge	16.53 (3.40)	11.40 (3.25)	1603.50
Pragma	11.98 (2.71)	5.07 (2.27)	1555.50
Mania	13.58 (4.26)	12.82 (1.93)	1672.00
Agape	8.42 (2.94)	7.82 (3.31)	1484.00
8. Our friendship merged gradually into love over time.	4.42 (0.80)	3.77 (1.30)	1218.00 *
17. I cannot be happy unless I place my partner’s happiness before my own.	3.42 (1.39)	2.87 (1.28)	1283.00 *
Sexual assertiveness in the use of contraceptive methods	2.69 (3.46)	5.03 (4.69)	1189.00 **

** *p* < 0.01; * *p* < 0.05. Regarding the Love Attitudes Scale, only items showing statistical significance are shown.

## Data Availability

The data presented in this study are available on request from the first author.
